# Improving the specificity of high-throughput ortholog prediction

**DOI:** 10.1186/1471-2105-7-270

**Published:** 2006-05-28

**Authors:** Debra L Fulton, Yvonne Y Li, Matthew R Laird, Benjamin GS Horsman, Fiona M Roche, Fiona SL Brinkman

**Affiliations:** 1 Department of Molecular Biology and Biochemistry, Simon Fraser University, Burnaby, BC, Canada; 2 Department of Medical Genetics, University of British Columbia, Vancouver, BC, Canada; 3 Canada's Michael Smith Genome Sciences Centre, 570 W. 7th Avenue, Vancouver, BC, Canada

## Abstract

**Background:**

Orthologs (genes that have diverged after a speciation event) tend to have similar function, and so their prediction has become an important component of comparative genomics and genome annotation. The gold standard phylogenetic analysis approach of comparing available organismal phylogeny to gene phylogeny is not easily automated for genome-wide analysis; therefore, ortholog prediction for large genome-scale datasets is typically performed using a reciprocal-best-BLAST-hits (RBH) approach. One problem with RBH is that it will incorrectly predict a paralog as an ortholog when incomplete genome sequences or gene loss is involved. In addition, there is an increasing interest in identifying orthologs most likely to have retained similar function.

**Results:**

To address these issues, we present here a high-throughput computational method named Ortholuge that further evaluates previously predicted orthologs (including those predicted using an RBH-based approach) – identifying which orthologs most closely reflect species divergence and may more likely have similar function. Ortholuge analyzes phylogenetic distance ratios involving two comparison species and an outgroup species, noting cases where relative gene divergence is atypical. It also identifies some cases of gene duplication after species divergence. Through simulations of incomplete genome data/gene loss, we show that the vast majority of genes falsely predicted as orthologs by an RBH-based method can be identified. Ortholuge was then used to estimate the number of false-positives (predominantly paralogs) in selected RBH-predicted ortholog datasets, identifying approximately 10% paralogs in a eukaryotic data set (mouse-rat comparison) and 5% in a bacterial data set (*Pseudomonas *putida – *Pseudomonas syringae *species comparison). Higher quality (more precise) datasets of orthologs, which we term "ssd-orthologs" (supporting-species-divergence-orthologs), were also constructed. These datasets, as well as Ortholuge software that may be used to characterize other species' datasets, are available at  (software under GNU General Public License).

**Conclusion:**

The Ortholuge method reported here appears to significantly improve the specificity (precision) of high-throughput ortholog prediction for both bacterial and eukaryotic species. This method, and its associated software, will aid those performing various comparative genomics-based analyses, such as the prediction of conserved regulatory elements upstream of orthologous genes.

## Background

Ortholog prediction is an important facet of comparative genomics and is frequently used in genome annotation, gene function characterization, evolutionary genomics, and in the identification of conserved regulatory elements. As the number of genome sequences grow, comparative genomics has become increasingly relevant. Errors in ortholog prediction can greatly affect such studies and associated downstream analyses (including functional genomics and proteomics analyses), so there has been increasing interest in high quality ortholog prediction.

Orthologs are commonly defined as genes that have diverged after a speciation event [1], whereas genes that have diverged after a gene duplication event, either before a speciation event (out-paralogs) or after a speciation event (in-paralogs), are collectively known as paralogs. It has been found that orthologs tend to have similar function and so their utility in comparative analyses is paramount. Classically, orthologous genes are identified by phylogenetic analysis. A phylogenetic tree for the genes is compared against a reference species tree, with the notion that the gene tree of orthologs should be similar to the species tree. However, sophisticated phylogenetic analysis is not easily automated, due in part to the complexity of both manual sequence alignment editing and choice of appropriate genes and species to be included in an analysis.

**Figure 1:**
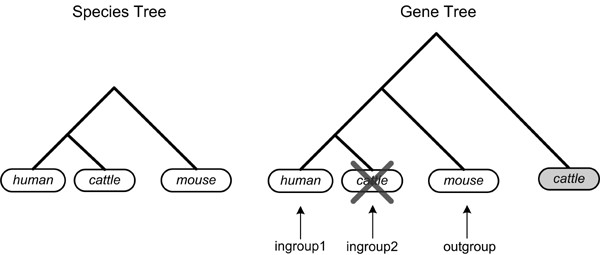
An example of how RBH analysis may falsely identify a paralog as an ortholog. Illustrated is a hypothetical species tree and gene tree for the human, cattle, and mouse species, where human and cattle orthologs (unshaded genes) are being identified. If the true cattle ortholog has not yet been sequenced because of an incomplete bovine genome project, it will not be present in the gene dataset used for analysis (cattle gene crossed out with an X), and the best reciprocal BLAST hit for the human gene will be a cattle paralog (shaded gene). However, Ortholuge will detect this case as a potential paralog, because it examines the relative phylogenetic distance between genes and identifies how well their relative distances match expected species divergence.

Whole-genome analyses indicate that many gene families (essentially paralogs) were formed before the divergence of most species commonly being compared in a comparative genomics analysis (out-paralogs). Therefore, orthologs – which diverged due to speciation – are typically more similar to each other than to other genes in the genome. This is why sequence similarity is often used to infer gene orthology between two or more species, and is also the premise behind the most common high-throughput ortholog prediction method used today: the reciprocal-best-BLAST-hits (RBH) analysis [2]. With the RBH method, genes from species A and species B are predicted to be orthologs if they are both the "best BLAST hit" of the other, when all genes from species A are compared to all genes from species B by BLAST analysis. There are numerous resources and methods that use a version of RBH as part of their ortholog prediction process, including the Clusters of Orthologous Groups (COG) database [3,4], The Institute for Genomic Research (TIGR)'s EGO database [5], and INPARANOID [6,7]. However, if a gene is not present in one organism's gene dataset, perhaps due to incomplete genome sequence data or gene loss in the organism, the RBH method will incorrectly predict a paralog as an ortholog (Fig. 1). Today, comparative genomics is often being performed using incomplete genomes, especially for large eukaryotic genome sequencing projects. Also, gene loss is a major driving force behind bacterial evolution [8]. It is therefore important to recognize that many of the current ortholog databases will likely contain false-positives due to the limitations of the RBH approach.

For comparative analyses, it is also frequently desirable to identify orthologs that most likely have similar function. In some cases, an ortholog may diverge more rapidly in sequence (and function) in one organism/species versus another related organism/species. In addition, a gene duplication may occur in one species, but not a second species, after species divergence. In this case either one – or both – of the duplicated genes (in-paralogs) are more likely to have diverged in function [9]. We therefore propose to differentiate such orthologs (reflecting what has sometimes been referred to as "many-to-many" ortholog relationships) from those that appear to have diverged *only *due to a speciation event. We also wish to identify those orthologs that have diverged to a degree that is similar to that expected for its species, since those orthologs that have undergone unusually rapid divergence in one species, relative to another, may have also diverged more in function. We therefore propose the term ssd-orthologs (for "supporting-species-divergence" orthologs) to define orthologs that appear to have diverged only due to speciation – and have diverged to the same relative degree as their species. These ssd-orthologs are more likely to have retained similar function, and would better suit the purposes of many comparative analyses. To avoid the confusion that may stem from the association of the term "many-to-many orthologs" with in-paralogs, we will use the term paralogs in this text to refer to out-paralogs and specify in-paralogs, when applicable.

To address these issues, we have developed a method we call Ortholuge. Ortholuge is a high-throughput analysis pipeline that evaluates previously predicted orthologs (such as RBH-predicted orthologs on a genome-wide scale) and generates predictions regarding which of these are likely ssd-orthologs and which are likely paralogs or other non-ssd-orthologs. The pipeline requires tentative ortholog predictions (and the associated gene/protein sequences) for large gene datasets from three species, two of which are the species to be compared, and one of which is an outgroup species. All phylogenetic distances between the genes/proteins in an ortholog group are computed for each group in the input list. Ratios of these distances are used to evaluate ortholog quality. We find that these ratios show certain consistencies over several sets of eukaryotic and bacterial orthologs, along with data sets introduced with true-negatives for comparison. This permitted the formulation of ratio cut-offs for retaining ssd-orthologs and removing probable paralogs, which resulted in a higher quality data set of orthologs. Overall, we demonstrate that the relative evolutionary relationships may be used to support the prediction of orthologs. In addition, noting those orthologs with prominent differences (such as recent gene duplications after species divergence) may help refine analyses to permit the identification of those orthologs that most likely retain the same function.

## Results

**Figure 2:**
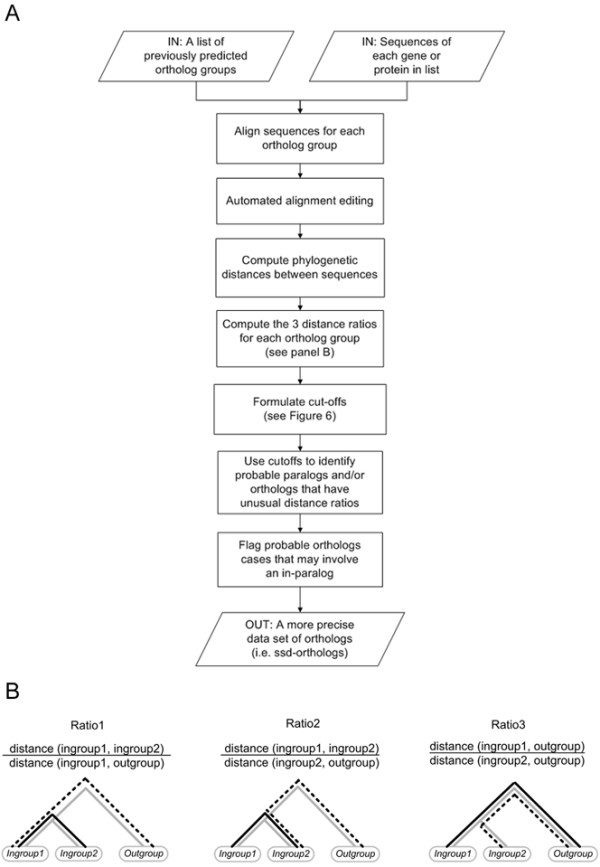
An overview of the Ortholuge method. (A) Flow-chart outlining the main steps of the method. (B) The three ratios computed by Ortholuge. The phylogenetic distances in the numerator (dark line) and denominator (dashed line) for each ratio is shown, overlaid on the phylogenetic tree (gray line) that relates the ingroups and outgroup. Note that the three ratios are related such that Ratio2 = Ratio1 × Ratio3. Therefore, ratio data is presented both in terms of frequency histograms for all three ratios (see Fig. 4) and also as Ratio1 × Ratio2 plots (see Fig. 5) for just two of the three ratios – the latter is simply another way to conveniently visualize the data.

An overview of the Ortholuge approach for increasing the specificity of ortholog predictions is outlined in Figure 2. Based on the analyses described below, the details of this approach were formulated and the approach validated using both prokaryotic and eukaryotic data sets. Ortholuge software is available [28] to assist with the analysis of data sets other than those reported here.

### Data sets exhibited little bias due the automated sequence alignment trimming approach

We investigated the behaviour and utility of Ortholuge through analysis of diverse eukaryotic and bacterial RBH-derived datasets. For the initial test eukaryotic data set, we chose predicted mouse-rat-human orthologs from the expressed sequence tag (EST) data in TIGR's Eukaryotic Gene Ortholog (EGO) database [5] (for a mouse-rat comparison, with human as the outgroup). The majority of our subsequent analyses utilized the higher quality MGD-based dataset (see Methods describing datasets) and the RefSeq-based RBH dataset composed of these same species, as indicated. For the bacterial data set, we chose three gamma-proteobacteria: *Escherichia coli*, *Pseudomonas putida*, and *Pseudomonas syringae *(a *Pseudomonas *species comparison, with *E. coli *as the outgroup). Orthologs between these three species (and other sets of species subsequently examined) were predicted using a transitive RBH approach, applied to the deduced proteins from complete genome sequences [10-12].

**Figure 3:**
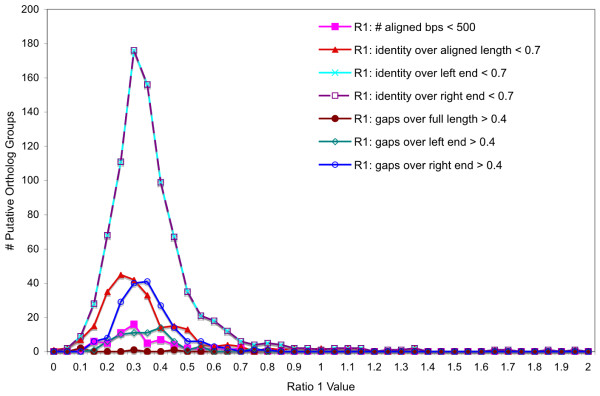
Ratio 1 (R1) ratio distribution curves for selected alignment characteristics. Higher quality mouse-rat-human ortholog sequence sets were analyzed to devise the gap-masking and sequence trimming approaches. These methods were evaluated for the introduction of ratio distribution biases for selected alignment characteristics such as identity and gap length. Ratio distribution curves were plotted for several characteristics. No obvious bias was observed through the introduction of our gap masking approach or alignment trimming.

Accurate sequence alignment is critical for phylogenetic analysis; thus, we wished to improve the automated alignment and trimming components of the Ortholuge method. We therefore performed a comprehensive examination of biases in our automated alignment editing process (see Methods). A sample of RBH-predicted ortholog sequence sets was analyzed to devise the gap-masking and sequence trimming approaches. The sequence sets were examined to identify both gaps introduced by misalignments and gaps introduced through sequence insertions and deletions. Our observations suggested that some of the noise introduced through the misalignment may be alleviated through the removal of the gapped-segment flanking portions. We also noted that there was no appreciable effect on the sequence distances when the flanking sequences around the sequence-variation gapped regions were removed. We manually introduced gap-masking simulations over the sequences using various window length criteria to establish a gap-masking approach with a relatively conservative worst-case scenario. Both the trimming and gap-masking methods were evaluated for the introduction of ratio distribution biases by selected alignment characteristics. No obvious bias was observed through the introduction of our gap masking approach or alignment trimming (Fig. 3).

### Ortholuge produces ratios which form distributions

**Figure 4:**
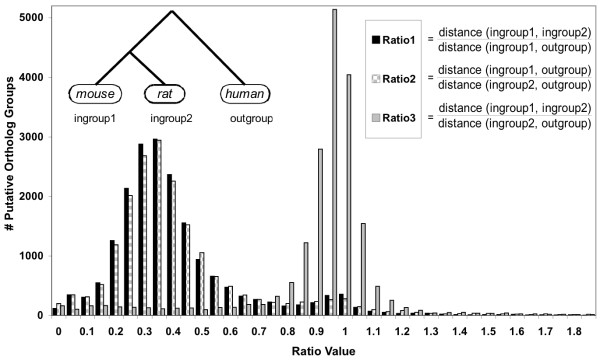
Histogram illustrating the distribution of RBH-predicted (i.e. putative) orthologous groups across the three Ortholuge distance ratios. The results for predicted mouse-rat-human RBH ortholog sets (EGO RBH data set; 19,200 ortholog groups) are shown. Each of the three ratios forms their own distribution: Ratio1 and Ratio2 are generally located at ratio values lower than 1 and Ratio3 is generally located about a ratio value of 1, reflecting the relative distances between ingroups and between each ingroup and the outgroup. A similar ratio analysis was performed on a RefSeq RBH dataset (see Figure 3 of [Additional file 1]).

Ortholuge was designed with the purpose of overcoming certain limitations of the RBH method, such as the problem illustrated in Figure 1. Ortholuge overcomes this problem by using ratios of phylogenetic distances between genes to evaluate orthology, and using an outgroup species as a reference for two ingroup species being compared (Fig. 2). For these three species, the distances for the "ortholog triple" are calculated and the three possible ratios that can be generated are calculated (Fig. 2). With this approach, the problem illustrated in Figure 1 would be detected because the human-cattle distance is unexpectedly larger than the human-mouse distance – impacting on ratio values. We ran Ortholuge on three mouse-rat-human datasets: two sets of RBH-predicted orthologs – one based on EGO data and the other based on RefSeq data – and a third high-quality curated set. For all datasets, human was the outgroup used to help predict more precise orthologs between mouse (ingroup1) and rat (ingroup2). The resulting Ortholuge phylogenetic distance ratios are shown in Figure 4 and Supplemental Figure 3 as histograms. For each of the three ratios, we tabulated the frequency of putative orthologous groups within certain ratio value ranges. Ratio1, Ratio2, and Ratio3 each form clear distributions. Ratio3 is generally located around a ratio value of 1, which is expected if the chosen outgroup is more distant relative to the ingroups. It is centered to the left or right of 1 depending on which of the two ingroups is closer to the outgroup. The Ratio1 and Ratio2 distributions are generally located at a ratio much lower than 1, reflecting the closer relationship between the ingroup species versus any ingroup to the outgroup. We ran our analyses on both protein and nucleotide sequences and found that for closely related species such as these, nucleotide sequences provide a better ratio distribution resolution. However, the overall ratio distributions are similar, even when using different methods of initial ortholog detection (see Figure 4 of [Additional file 1]).

We also performed this analysis with our bacterial *P. putida*-*P. syringae*-*E. coli *orthologs, comparing *P. putida *(ingroup1) and *P. syringae *(ingroup2) using *E. coli *as the outgroup. We observed very similar results: Both the eukaryotic and prokaryotic data sets are consistent in the distributions formed, and in the approximate position of the distributions. Since we expected most ssd-orthologs (see Introduction for definition) to evolve in a similar manner, we hypothesized that orthologs falling within the higher frequency ranges of the distributions are more likely to be ssd-orthologs compared to those that are outliers. In essence, what is defining the species divergence is the divergence observed for most genes (i.e. the highest frequency ranges).

### Ortholuge ratios can also be conveniently visualized in an R1 × R2 plot

Instead of histograms (Fig. 4), an alternative way to represent Ortholuge ratios is to use a 2-dimensional plot of two Ortholuge ratios, where each putative ortholog group is represented by one point in the graph. In principal, any two of the three ratios can be used for the plot, since the three ratios are related. That is, Ratio3 equals Ratio2 divided by Ratio1. Through subsequent analyses, we found that the Ratio1 and Ratio2 combination (i.e. an R1 × R2 plot) was the simplest to visualize and to work with.

**Figure 5:**
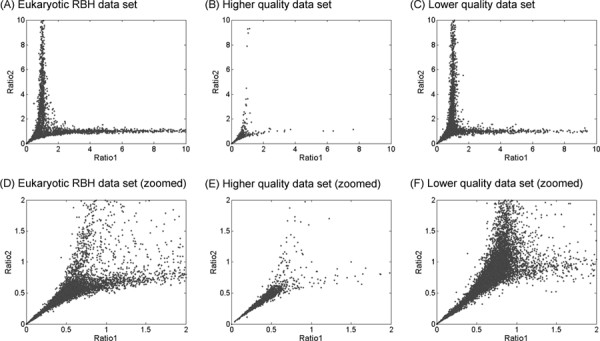
Ortholuge R1 × R2 plots (Ratio1 versus Ratio2) for selected eukaryotic data, where each point represents one putative ortholog group. (A) Putative orthologous groups identified using RBH for mouse-rat-human (Figure 4 shows the corresponding histogram). (B) Putative orthologs groups for mouse-rat-human from a higher quality (more precise) dataset (see Methods). It is expected that this more precise data set comprises primarily true orthologs. (C) A lower quality data set of RBH-predicted orthologous groups for cattle-human-mouse, where cattle genes have been identified from an incomplete genome sequence. (D), (E), (F) are zoomed-in versions of (A), (B), (C), respectively, with axes shown from 0 to 2 instead of 0 to 30. Note that most orthologous groups exhibit low Ratio1 and Ratio2 values, in all three data sets. For example, in panels A and D, about 86% of orthologs have Ratio1 and Ratio2 values less than 1. However, the higher quality data set (panels B and E) contains fewer points at higher Ratio values versus the RBH-predicted data set. The lower quality data set contains more points with very high Ratio2 values (i.e. only 73% of points have Ratio1 and Ratio2 values less than 1), potentially reflecting the increased occurrence of probable cattle paralogs (i.e. paralogs being misidentified as orthologs by an RBH-analysis with an incomplete cattle genome).

**Figure 6:**
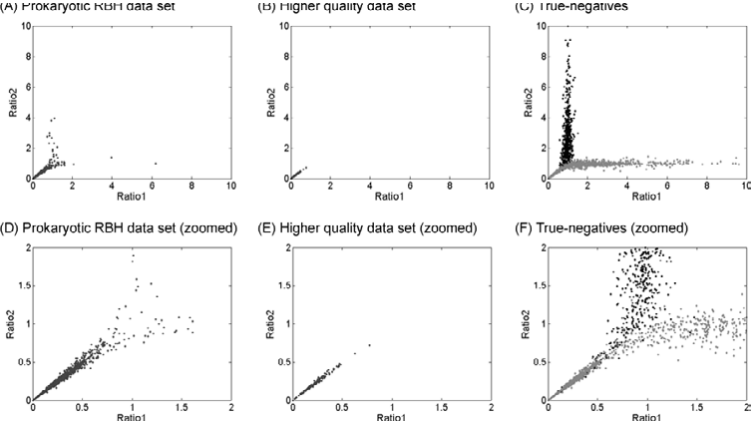
Ortholuge R1 × R2 plots for the prokaryotic data, illustrating two ortholog data sets and a true-negative data set. (A) Putative orthologous groups from an RBH-predicted data set. (B) Probable true orthologs from a higher quality (more precise) data set. (C) True-negative orthologs (i.e. true paralogs) from the "gene-loss simulation" data set. Darker dots represent putative orthologous groups which have had an ingroup1 true-negative (paralog) introduced into the group. Lighter dots represent putative orthologous groups which have had an ingroup2 true-negative (paralog) introduced into the group. (D), (E), (F) are zoomed-in versions of (A), (B), (C), respectively, with axes shown from 0 to 2 instead of 0 to 10. Most putative ortholog groups (particularly for the high quality data set) exhibit low Ratio1 and Ratio2 values (for example, all values are less than 1 for the points in the high quality data set plot), whereas most true-negative groups exhibit higher Ratio1 and Ratio2 values (i.e. only 9% of ingroup1 true negative introductions, and 6% of ingroup2 true negative introductions, have points with Ratio1 and Ratio2 values less than 1).

For the R1 × R2 plots, the eukaryotic mouse-rat-human RBH-predicted putative orthologous groups appear to occupy three types of positions (Fig. 5A and 5D). (1) The majority of points form a cluster (highest frequency range) at low Ratio1 and Ratio2 values. In fact, about 85% of orthologs have Ratio1 and Ratio2 values less than 1. (2) Some points with higher Ratio1 values are located along a curve that approaches, and then falls along, the line equation Ratio2 = 1. This is consistent with an unusually high divergence of a gene from ingroup 2. (3) Conversely, some points with higher Ratio2 values are located along a line that is roughly around line equation Ratio1 = 1. This is consistent with an unusually high divergence for a gene from ingroup 1. The RBH-predicted orthologous groups for *P. putida*-*P. syringae-E. coli *species show a similar R1 × R2 plot (Fig. 6A and 6D). Consistent with the eukaryotic results, the vast majority of orthologous groups for this prokaryotic analysis also exhibit Ratio1 and Ratio2 values less than 1.

We expected most ssd-orthologs to evolve in a similar manner, and found that most orthologous groups form a cluster (high frequency range) in an R1 × R2 plot. Therefore, we hypothesized that orthologous groups falling within the high frequency range are more likely to contain ssd-orthologs. Conversely, those outside of this range (i.e. high Ratio1 or Ratio2 values) are more likely to contain, in an ingroup, either an ortholog that has undergone unusual divergence, or a paralog.

### "Higher quality" orthologous groups are found primarily in "low" Ortholuge ratio ranges, in R1 × R2 plots

The data sets of tentative orthologs predicted above by an RBH approach will certainly contain genes that are being falsely identified as orthologs. It is difficult, if not impossible, to obtain a dataset of this size that contains only true orthologs, due to the inherent nature of inference associated with evolutionary study. However, data sets of "higher" and "lower" quality can be constructed and examined (see Methods), to observe how their Ortholuge ratios change in comparison to each other. These data sets should contain a notably greater or smaller proportion of true orthologs, respectively.

We therefore examined the behaviour of Ortholuge ratios for a higher quality data set of probable orthologs. Curated orthologs between human, mouse, and rat genomes were acquired from the Mouse Genome Database (MGD). Figure 5B and 5E illustrate that this higher quality data set occupies a smaller area of the R1 × R2 plot. This smaller area is observed, even when the number of points is normalized with the number plotted for the RBH-based data (data not shown). For this higher quality (more precise) data set there are notably fewer points along the Ratio1 = 1 line equation and the Ratio2 = 1 line in the plot, compared to the RBH-based data plot in Figure 5A and 5D.

Conversely, we examined the ratios associated with a "lower quality" data set, involving RBH-predicted orthologs for bovine, human, and mouse, from TIGR's EGO database (with mouse as the outgroup). The incomplete state of the bovine genome data at the time of this analysis should lead to more falsely predicted orthologs, since some true orthologs will be missing from the bovine dataset (see Fig. 1 for a scenario). These results are shown in Figure 5C and 5F. Note the higher number of points with a high Ratio2 value, falling along the line equation Ratio1 = 1; these points are consistent with how the ratio would behave if the bovine data contained paralogs that were notably more divergent than expected for most orthologs.

To gain a sense of the differences in plots of different quality datasets, note that below Ratio1 and Ratio2 values of 1, there lies 97% of high quality dataset points (Fig. 5B), 86% of RBH-predicted ortholog group points (Fig. 5A), and only 73% of the low quality data set points (Fig. 5C). These results suggest that true orthologs (or at least more precise ortholog data sets) tend to fall within the bulk of the highest frequency range (i.e. relatively "low" Ratio values in an R1 × R2 plot), while orthologs with unusual divergence patterns (non-ssd-orthologs) and paralogs have either high Ratio1 or high Ratio2 values.

For the prokaryotic analysis, a higher quality data set was compared to the RBH-based data set as well. Figure 6A and 6B illustrate the same trend as the eukaryotic data, with respect to how the R1 × R2 plots look for more precise and less precise ortholog data sets.

### Known paralogs (true-negatives) introduced into orthologous groups generate either high Ratio1 or high Ratio2 values, as shown in a gene loss/incomplete genome simulation

The above comparisons of higher quality (more precise) and lower quality (less precise) ortholog data sets support our hypothesis that orthologs and paralogs fall within different regions of the R1 × R2 plot. However, a stronger argument can be made by examining specifically where falsely predicted orthologs (true paralogs) occur in such distributions. A true-negative data set was therefore constructed by removing genes from one of the ingroup gene data sets and then identifying the next best reciprocal BLAST hit with the other ingroup (ensuring transitivity of this introduction with the other ingroup and outgroup). Therefore a true negative is essentially an ortholog triple which has been transformed into a false positive by introducing a less similar sequence for one of the species sequences. These true negatives represent the types of ortholog predictions that would result from an RBH-method in scenarios such as Figure 1. Since we know that RBH can make incorrect predictions when a genome is incomplete or when gene loss has occurred, this analysis simulates what would occur with the RBH method in such cases. The benefit of this analysis is that we specifically know the true-negatives introduced, allowing us to examine how the Ortholuge ratios for these true-negatives (paralogs) behave.

For the *E. coli-P. putida-P. syringae *input ortholog groups, we constructed two true-negative data sets. In the first, we replaced *P. putida *genes with their next best RBH hit to *P. syringae*, resulting in ingroup1 paralogs. In the second, we replaced *P. syringae *genes with their next best RBH hit to *P. putida*, resulting in ingroup2 paralogs. For both, we conservatively introduced all possible paralogs into the analysis, resulting in roughly 50% of the genes converted to true-negatives (i.e. conservative, because most data sets would never contain this many true-negatives). The results from these two data sets (Fig. 6C and 6F), show that these true-negatives overlap very little with the RBH-predicted orthologs (Fig. 6A) or with the high quality (more precise) orthologs (Fig. 6B). This demonstrates that even with all possible true paralogs simulated, very few of them are falling within the higher frequency ranges of the RBH distributions.

**Figure 7:**
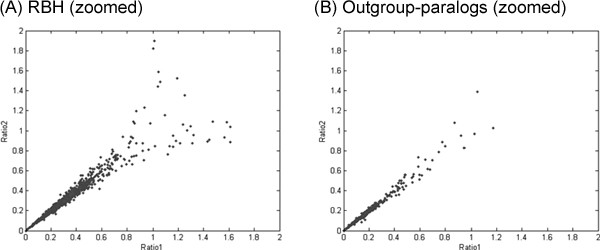
R1 × R2 plots, for the prokaryotic data, illustrating the effect of introducing outgroup paralogs (outgroup ortholog true-negatives) in the analysis. Unlike for other figures of R1 × R2 plots in the paper, only ratio ranges from 0 to 2 are shown for each axis. (A) RBH-predicted orthologous groups. (B) Outgroup paralogs from a true-negative data set where all possible outgroups were replaced with next best RBH paralogs. They cannot be well distinguished from other orthologs, however, this is actually promising, since Ortholuge is in essence identifying orthologs between the ingroups only. This analysis shows that an outgroup paralog does not interfere greatly with the identification of true orthologs shared between the ingroups.

We also constructed a third true-negative data set with all outgroup genes (*E. coli*) replaced by their next best RBH hit to both *P. syringae *and *P. putida*. The R1 × R2 plot (Figure 7) shows that these true-negative cases plot at lower Ratio1 and Ratio2 values and do not separate well from what would be expected for true-orthologs. This is actually promising, since in the case of a paralog in an outgroup, the two ingroups should still be regarded as probable true orthologs and should still be falling within the main cluster of true-orthologs, as we observe. In other words, since the goal of Ortholuge is to improve ortholog identification between the two ingroups, it is beneficial that an outgroup paralog does not generally interfere with/affect the analysis.

### Ortholuge ratio cut-offs, to separate orthologs from paralogs, can be determined based on an iterative-true-negative analysis

**Figure 8:**
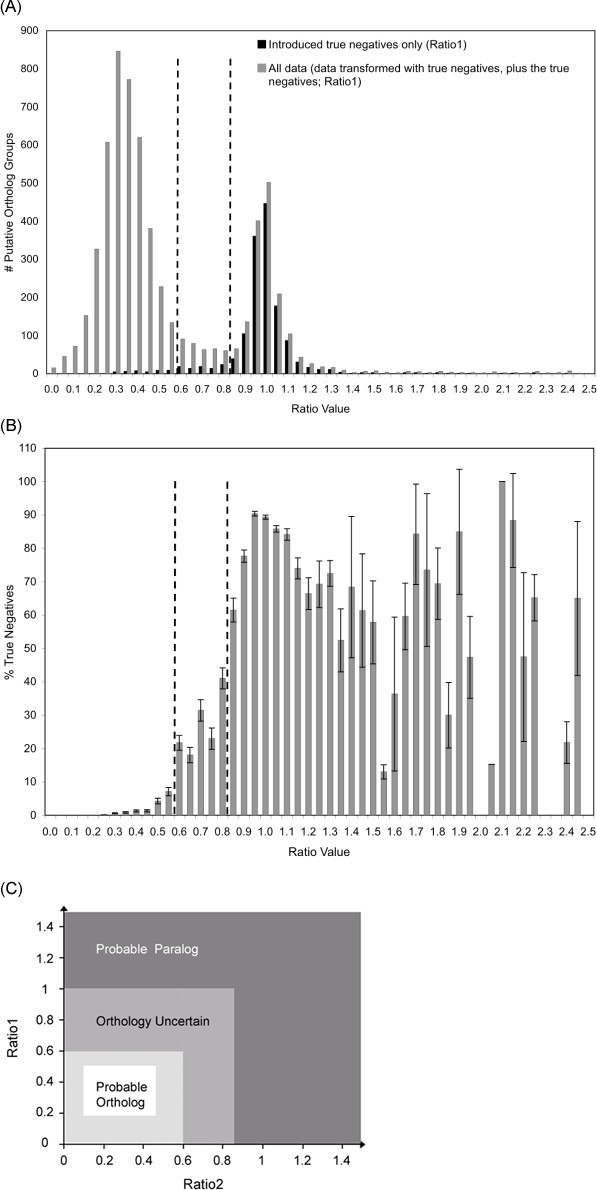
Example of the generation of cut-offs for classification of ssd-orthologs and probable paralogs, based on an iterative-true-negative analysis (i.e. based on an introduction of random sets of true-negatives). The particular analysis illustrated here is a Ratio1 analysis for the mouse, rat, human RefSeq RBH dataset, with true-negatives introduced into the mouse (ingroup1) set. In panel A, the number of putative orthologous groups in each ratio range for the true-negative-transformed data set is shown for the whole data set (light shaded bars) and for just the introduced true-negatives only (dark shaded bars). Note how the distribution of the data set differs from that of the true negatives (i.e. introduced paralogs). In panel B, the proportion of randomly introduced true-negatives at 0.5 ratio range intervals is used to formulate cut-offs (denoted by dashed lines) for classifying ssd-orthologs and probable paralogs for the analysis. For the ssd-orthologs cut-off (left-most dashed line), no more than 10% true negatives in a given ratio range are permitted for the ssd-orthologs range. For the probable paralogs cut-off (right-most dashed line) the proportion of true negatives is at or above 50 percent. The resulting middle region bounded by these two cut-off points establishes the "uncertain" orthology class ratio range. Dashed-lines denoting these particular cut-offs are also illustrated on the figure in Panel A for reference. This approach for a true-negative analysis and cut-off generation is also performed for Ratio2 [Additional file 1] and the combination of cut-offs for Ratio1 and Ratio2 are used to classify putative orthologous groups from another data set (such as an RBH-predicted data set) into the three classification levels of "probable ssd-ortholog", "uncertain" and "probable paralogs". Panel C schematically shows the areas of an R1 × R2 that would be classified in this way, with the cut-off numbers in this particular example matching the RefSeq RBH-based mouse-rat-human analysis (see Table 2 for how these ranges are numerically determined).

After determining that the introduced true-negatives almost never fall within certain ratio ranges, it became clear that ratio cut-offs could be derived to exclude most true-negatives, and thus improve the specificity (precision) of ortholog prediction. To do this, another strategy was employed to simulate the introduction of paralogs (true-negative ortholog predictions) and then formulate ortholog identification cut-offs. This second strategy, involving an iterative-true-negative analysis, allows one to view the variance in proportion of true-negatives in a particular ratio range, and is also amenable to high throughput use for the formulation of cut-offs. For both the eukaryotic (human-mouse-rat) RBH-predicted data set (RefSeq-based), and the prokaryotic RBH-predicted data set, we conservatively modeled an incomplete genome (or gene loss) scenario by randomly replacing 25% of the genes in the RBH-predicted data set with the "next best RBH" hit (i.e. a true-negative). This randomized introduction of true-negatives was iterated at least 50 times, and each iteration was evaluated by Ortholuge. The proportion of true-negative orthologs was averaged over all iterations and the standard deviation determined. We found that that once again, the ratio values of true-negative orthologs do not overlap well with those of the bulk of RBH-predicted orthologous groups (Figure 8 and Supplemental Figures 1 and 2).

For both the prokaryotic and eukaryotic RBH-based data sets, this iterative true-negative analysis was used to determine ratio ranges where true paralogs were very unlikely to land and ranges where they were very likely to land. The borders of these ranges (described in Figure 8 and Supplemental Figures 1 and 2) became the ratio cut-off values. This permitted classification of the RBH-predicted tentative orthologous groups into probable ssd-orthologs, probable paralogs, or "uncertain" categories. It should be noted that a more accurate name for the 'probable paralog' category might be 'probable non-ssd-ortholog,' because there may be true orthologs that have undergone unusual divergence in one ingroup species within this category. However, in such cases the non-ssd-orthologs may have functionally diverged, and therefore are cases that we would want to differentiate from our ssd-ortholog set. Regardless, for ease of comprehension, we propose to call those cases with very atypical ratios (in the range of what is observed for paralogs) "probable paralogs", since paralogs likely predominate in this region.

We chose a 25% true-negative introduction, since this is likely above a worst-case scenario in terms of the number of genes that may be missing in an incomplete genome, or most cases of naturally occurring gene loss. We felt it was important to "saturate" the data set with true-negatives, because any given RBH-based dataset will likely contain some proportion of false-positives in the putative orthologous groups (i.e. it is difficult to ensure one has a completely true-positive set of orthologs). Therefore, to effectively identify the ranges where true-negatives were becoming increasingly more common we needed to observe a large proportion of true-negatives. However, we did not want to transform a data set with all possible true-negatives, as this would not provide a sense of the variation in proportion of true-negatives within a given ratio range. Note that we also chose to report the results here for a transformation of an RBH-predicted data set with the true-negatives (i.e. a RefSeq-based RBH analysis), rather than a transforming a high quality dataset, since the RefSeq based analysis could be more easily fully automated (i.e. it did not require developing a curated set of high quality orthologs). However, transformation of a eukaryotic high quality dataset with true-negatives generated similar cut-off values (data not shown). Through an iterative sampling approach we were able to generate standard deviations of the proportion of true-negatives in a given ratio range (Figure 8B), providing a clearer picture of the likelihood of a true-negative occurring in that range.

### Ortholuge ratios in combination can help predict which gene in a given putative orthologous group is likely a paralog

A closer inspection of the Ortholuge ratios shows that they behave in a predictable fashion when the ortholog group contains one or more false-positives (Table 1). For example, if ingroup1 is actually a paralog, then the distance between ingroup1-outgroup and the distance between ingroup1-ingroup2 would be larger than the norm for an ssd-ortholog. This would cause Ratio2 to increase (the degree of increase would depend on how diverged the paralog is from the missing 'true' ortholog), and Ratio1 to increase a slighter amount (depending on how distant the outgroup is). Conversely, if ingroup2 is actually the paralog, then Ratio1 would be expected to increase and Ratio2 to increase slightly. These predictable changes do indeed occur, as illustrated by an analysis of true-negatives (Figure 6C and 6F), an analysis of a dataset of tentative orthologs identified by RBH using an incomplete genome (Figure 5C and 5F), and an additional manual review of selected cases (data not shown). We propose that when unusual ratio ranges are identified for a given orthologous group, the relative changes can facilitate predictions regarding which of the two ingroups may contain a paralog (or non-ssd-ortholog).

Note that an outgroup paralog cannot be well predicted, however this does not affect the utility of Ortholuge, since the method is focused on characterizing the orthology of the two ingroups. It should also be noted that multiple-paralog scenarios (last three rows in Table 1), are more complex. Though relatively easy to predict on paper, they are more difficult to distinguish in reality, because the amount of divergence for the two paralogs may vary greatly. In most cases they would resemble one of the first three scenarios, depending on which of the two paralogs was more diverged. Nevertheless, in the end, these rare cases (two paralogs in a group of three) will still most frequently display atypical ratios, and will not fall within probable ortholog cut-offs.

### Ortholuge in action: an estimation of probable ssd-orthologs and probable paralogs in RBH-based data sets

An example of ratio cut-offs generated based on our true-negative analysis is listed in Table 2 (see also Figure 8 and Supplemental Figures 1 and 2). Researchers are of course encouraged to choose their own cut-off to suit their needs (i.e. more sensitivity or specificity). However, based on our simulations, these cut-offs should effectively differentiate probable orthologs and paralogs for these data sets. We also propose that these cut-offs can identify those orthologs most closely following species divergence (i.e. ssd-orthologs) – orthologs which may be more functionally similar to each other versus those that have diverged at different evolutionary rates in each species.

Using the derived ratio cut-offs, we have constructed several data sets of probable ssd-orthologs consisting of: mouse-rat comparisons (with human as the outgroup), and one for a *P. putida*-*P. syringae *comparison (with *E. coli *as the outgroup). These ssd-orthologs are particularly suited for comparative genomics analyses. In addition, notations are added to all the data analysed, indicating cases of probable gene duplication after species divergence ("possible in-paralog") – a scenario that can increase the likelihood of functional divergence of the genes. These higher quality sets of orthologs can be found via the Ortholuge website [28]. The proportion of ssd-orthologs in the RBH-predicted data sets is summarized in Table 2. Note that cases of in-paralogs are not counted within the counts of ssd-orthologs in Table 2. Such cases, due to their uncertain potential to have diverged in function because of a gene duplication, are counted within the "uncertain" category.

Using the cut-offs, we were also able to estimate the proportion of RBH-predicted orthologs that are likely paralogs for these eukaryotic and prokaryotic data sets (Table 2; see also data available on the Ortholuge website [28], which includes a classification of the EGO dataset using the RefSeq analysis cut-offs). For the prokaryotic data about 5% of RBH-based predictions are probable paralogs. For the eukaryotic data, about 10% of the RBH-predictions are probable paralogs. These are significant numbers that validate the need for a method like Ortholuge, particularly if one is trying to use RBH-predicted orthologs for downstream analyses that require stringent ortholog prediction (for example, for regulatory element detection).

Application of these cut-offs to classify the curated eukaryote and prokaryote datasets suggest that the false negative rate in is in the range of 0.7% for prokaryote data and 3% for the eukaryote data.

To facilitate the analysis of other datasets, we have developed Ortholuge software that can be used to characterize any existing dataset of orthologs. If no pre-existing ortholog dataset is available, Ortholuge can also construct such a dataset using an RBH-based approach applied to whole genome datasets (or other adequate datasets of genes from three organisms that a user supplies). Ortholuge was developed using Perl under Linux (SuSE 9.0 and RH 9.0) and operates in any UNIX environment, provided all the needed tools (see Methods) are available for the user's operating system. This freely available, open source, software is available on the Ortholuge website [28].

## Discussion

For cross-genome comparison purposes, researchers often wish to compare orthologs – in particular orthologs that have not undergone unusual divergence rates relative to one another, and have more likely retained similar function. We propose that Ortholuge is an approach, suitable for high-throughput genome-scale analysis, which aids identification of such orthologs. The Ortholuge method significantly improves the specificity/precision of high-throughput RBH-based ortholog analysis. For example, our results indicate that roughly 1 in 10 RBH-predicted rat-mouse orthologs are very likely paralogs, and about 1 in 20 RBH-predicted orthologs for two *Pseudomonas *species are similarly likely incorrect. Note that our RBH analysis requires transitivity between three species, rendering it more stringent than the typical RBH analysis between two species. This suggests that the typical RBH analysis may have an even greater number of false predictions. The resulting more specific identification of orthologs by Ortholuge is an important requirement for many downstream analyses, such as identifying gene regulatory regions, or characterizing differences in microarray-measured gene expression responses across species. An automated method such as Ortholuge is of course no substitute for a more manual, comprehensive phylogenetic analysis and has some limitations as mentioned below. However, its simplicity and utility for high-throughput analyses suggest that it is a useful complement to RBH-based identification of putative orthologs using whole-genome gene datasets. In addition, Ortholuge's higher specificity approach can complement other methods that may provide a higher sensitivity/recall approach for ortholog identification [13].

Ortholuge evaluates orthologs through phylogenetic distance comparisons. To perform such comparisons, an outgroup is required to assist the prediction of orthologs between the two ingroups – this has simultaneous advantages and disadvantages. The added sequence provides extra resolution and extra specificity; however, a distant outgroup may lessen the sensitivity of the approach. Presumably, though, as more genomes are sequenced, the number of possible outgroups available to choose from will increase and very distant outgroups will become less of a problem.

The Ortholuge pipeline generates predictions by evaluating the entire genome at once (or at least adequate gene representation for the species). The more data points that are representative of the genome, the more confident the ratio cut-offs will be. It assumes that the majority of incoming predictions are true orthologs, will exhibit expected ratios, and will thus form the high frequency ranges of the distributions. Our analysis does suggest this assumption to be reasonable and, notably, both eukaryotic and bacterial orthologs display similar ratio distributions, despite marked differences regarding how such organisms evolve.

Once the genome-wide predictions are made for a certain species combination, Ortholuge can be used to estimate how likely it is that a specific putative orthologous group contains a true-negative within its ingroups. In such cases, we can match these ratios with a category (i.e. classification shown in Table 2), to suggest which gene in the ortholog group is likely to be the paralog. However, it should be emphasized that at this time we have not exhaustively examined all possible scenarios, and so such analysis should be taken as a guide requiring further investigation. Interestingly, this method also appears to be useful to examine, in a genome-wide scale, the relationships between species. By examining the ratio values at the highest frequency ranges in the histograms, one can easily determine which two of any three organisms are more similar to each other, on average, and on a genome-wide scale (for example, that cattle genes are more similar to human genes, than mouse genes are to human genes, on average).

The simplicity of Ortholuge allows for many benefits. For example, it can easily be re-run when genome annotations undergo significant changes. In addition, it can easily be customized with any method of sequence alignment or phylogenetic distance calculation, depending upon the researcher's preference. It is expected that further analysis will reveal relationships between true-negative analyses and ratio cut-off generation, negating the need to perform a full iterative-introduced-true-negative analysis for each species comparison. Of course, users can choose their own Ortholuge ratio cut-offs, either using a true-negative analysis, or another approach of their choice, for identification of orthologs at their preferred level of specificity.

Accepting only orthologs in a certain ratio range and discarding the rest will certainly eliminate a small fraction of true orthologs from the input set. For example, if the probable paralog cut-offs are applied to the "high quality" curated prokaryotic and eukaryotic data sets, we eliminate 0.7% and 3% of the prokaryotic and eukaryotic predictions, respectively. However, if the more stringent ssd-ortholog cut-offs are applied, we eliminate 1.4% and 8% of the predictions, respectively. While these outliers may be false-positives in the curated data, they may also be true orthologs that have undergone unusual divergence in one ingroup species. For example, if a gene duplication occurred in one ingroup species after the speciation divergence, the resulting duplicated gene may undergo accelerated evolution [14]. Such scenarios would result in skewed ratios for true orthologs. However, we propose that such orthologs with unusual (relative) divergence may more likely have differing function at some level. In many genome-wide studies involving comparisons between species, researchers wish to identify those genes that are more likely to be functionally equivalent – i.e. orthologs that did not experience unusual rates of evolution or gene transfer. Ortholuge improves the identification of such "supporting species divergence" ortholog pairs (i.e. ssd-orthologs).

This is apparently an important issue, as illustrated by some confusion occurring in the literature regarding the definition of orthologs. The definition that we, and many evolutionary biologists use, is the one initially proposed [1] that describes orthologs as genes that have diverged due to speciation (rather than due to gene duplication, which describes paralogs). However, the term ortholog is increasingly being inferred to mean 'functionally equivalent genes in different species' – a common misconception [15]. While we and others agree that orthologs *tend *to have similar function, this is not a requirement for orthology [16]. So, it appears that while many researchers are identifying orthologs in a genetic or genomic study, what they really wish to identify is the subset of orthologs that are specifically functionally equivalent.

Some methods, such as the widely used INPARANOID, refer to all in-paralogs (i.e. genes created by gene duplication after the species divergence) in the one species as orthologous to the related gene in the other species. They do not clearly distinguish between such cases of in-parology and more simple one-to-one orthologous relationships. We believe that such cases should be differentiated because a duplication event after species divergence may have led to significant functional divergence of one or both of the duplicated genes in the one species. In Ortholuge, cases involving possible in-paralogs are flagged using a simple analysis that focuses on detecting the most clear-cut in-paralog cases. For our analysis, we did apply ratio cutoffs derived using one-to-one ortholog RBH-based (RefSeq) data to classify a same species (EGO) data set that includes both one-to-one orthologs and many-to-many orthologs. However, we recognize a need to implement more robust procedures that would consider all cases of suspected recent gene duplications in the analysis (the current method is subject to the limitations of the initial RBH-based ortholog identification). It would also be desirable to complement this analysis further by noting cases of relative gene rearrangement in the input set of orthologs. Ortholuge in its current form cannot detect gene rearrangements, however it could potentially complement other bioinformatics approaches that detect such rearrangements [17]. Ortholuge could also be adapted to contain a gene rearrangement analysis that is customized to its methodology. These additional ortholog evolutionary scenarios, involving possible in-paralogy or gene rearrangements, should be specifically noted because they cannot be distinguished by examining Ortholuge distance ratios alone. They require further study in any comparative analysis, since functional equivalence between the orthologs is less likely.

Regarding the limitations of this method, it should be emphasized that Ortholuge is limited by the quality of the initial ortholog-analysis (i.e. RBH can miss cases of true-orthology, and some data sets such as those from EGO are incomplete and don't clarify which genes are isoforms, which complicates in-paralog analysis). Ortholuge is also only as good as the quality of the sequence data being analyzed. We have tested our alignment trimming and masking of regions of lower alignment quality extensively to improve the critical sequence alignment component of our method; however, certainly this method will fail if low quality sequences, with many errors, are used in the analysis. In addition, the top BLAST hit is not necessarily the nearest neighbour [18] and so true orthologs may be missed when using Ortholuge after initially identifying orthologs with an RBH-based approach. Ortholuge could therefore improve if the initial ortholog prediction method is improved (it should be emphasized that Ortholuge can be used with any input dataset of proposed orthologs deduced by any current or future ortholog prediction methodology – not just the ones presented).

Regardless of any limitations, Ortholuge appears to effectively improve the specificity of ortholog identification and is suitable for high-throughput, genome-wide use. Given the amount of genomics data being obtained at this time, such specific, high-throughput approaches will become increasingly necessary, as genomics research moves further toward more multi-genome comparative analyses.

## Conclusion

Ortholuge improves the specificity of ortholog identification and is suitable for high-throughput use. This precise ortholog prediction method complements other ortholog prediction methods that are not focused on precision and it potentially identifies those orthologs most likely to be functionally similar. The Ortholuge method provides important data set evaluation for a variety of analyses based on comparative approaches, including gene function prediction, prediction of conserved regulatory elements, and comparative analysis of gene order or gene/protein expression data.

## Methods

### Data sets

#### 1. Eukaryotic Gene Orthologs (EGO) RBH data set

EGO release 8 database was obtained from The Institute of Genomic Research (TIGR) [5]. This database is composed of two files: 1) one file housing ortholog identifiers and tentative consensus sequence (TC) identifiers and 2) a second file TC sequences in FASTA format. Both files were used to extract and create 19,200 unique mouse, rat, human tentative ortholog gene sets files (TOGs) for Ortholuge analysis.

#### 2. Eukaryotic curated orthologs ("high quality" MGD dataset)

The Mouse Genome Database (MGD) is a comprehensive, high-quality database which currently includes orthology information for mouse, human, rat, and 14 other mammals [19]. Orthology annotations are manually curated from scientific literature and each orthology assertion is based on criteria recommended by the Human Genome Organisation (HUGO).

A program was developed to extract the orthologous gene pairs from the MGD Sybase database for two species triples: 1) mouse, rat, human 2) cattle, human, mouse. All relevant human, mouse, and rat RefSeq [20] mRNA sequences and protein sequences were obtained from the National Center for Biotechnology Information (NCBI) FTP site along with the Locus Link RefSeq mappings file. FASTA-formatted ortholog sets for those ortholog pairs were created that satisfied a transitive, triple ortholog relationship and had corresponding RefSeq sequences annotated with a *reviewed *or *validated *status. 2642 mouse, rat, human mRNA, 2499 mouse, rat, human protein, and 427 cattle, human, mouse mRNA ortholog sets were created.

#### 3. Eukaryotic Gene Orthologs (EGO) "lower quality" RBH set

Cattle, human, and mouse ortholog groups, totaling 16,134 in number, were extracted from the EGO release 8 database. The cattle genome was incomplete at this time and thus we expected more incorrectly predicted orthologs by the RBH method (see Fig. 1 for the scenario).

#### 4. Eukaryotic RefSeq-based RBH ortholog set

The species-specific mouse, rat, human RefSeq files were obtained from the NCBI FTP site and BLAST databases [2] were constructed for each file. A pairwise blastall analysis was performed between each species enforcing a 10e-04 E value cut-off. 6294 ortholog FASTA-formatted sets were created from transitive, best-hit mRNA RefSeqs. We allowed one unique best-hit isoform per Locuslink ID in the RBH dataset.

#### 5. Eukaryotic RBH Tentative Consensus (TC) ortholog set involving cattle

A higher-quality, non-redundant RBH TC dataset was established using the cattle, human, and mouse tentative sequences found in the EGO release 8 database. The transitive, triple reciprocal top best BLAST hit for each unique cattle TC was used to form 15,660 ortholog groups. This approach served to reduce the over-representation of TC's found in the currently established set of EGO tentative ortholog groups (TOGs) due to the allowance of multiple RBH relationships within a specified cut-off.

#### 6. Bacterial RBH-predicted data sets

Protein sequences of *Escherichia coli *K12 [10], *Pseudomonas syringae *pv. tomato str. DC3000 [11], and *Pseudomonas putida *KT2440 [12] were obtained from NCBI. For the RBH analysis, first a BLASTp was performed between all pair-wise combinations, with an E-value cut-off of 10e-04. Genes that retained a transitive reciprocal best hit property and passed the BLAST cut-off were retained. There were 1456 ortholog groups constructed.

#### 7. Bacterial higher quality orthologs

A set of higher quality orthologs was constructed from a set provided by Lerat et al [21], who found all the gene families in 13 gamma-proteobacteria genomes that had exactly one gene per species. For simplicity, we chose those that had annotated gene names in each of our three chosen bacterial species. Initially, there were 156 ortholog groups, and of these 143 ortholog groups passed our automated alignment editing stage.

#### 8. OrthoMCL eukaryote ortholog dataset

The OrthoMCL database files were downloaded [22] and a set of mouse-rat-human ortholog triples were extracted from the OrthoMCL clusters to construct ortholog triples. These predicted ortholog groups were analyzed using the Ortholuge analysis software.

Through our analyses, we observed that the use of nucleotide sequences provided better resolution for these particular sets of eukaryotic data, at the level of divergence being examined using Ortholuge (see Figure 4 of [Additional file 1]), whereas protein sequences provided better resolution for the particular bacterial data we were analyzing (data not shown). Consequently, all analyses below were performed using nucleotide sequences for eukaryote analysis and protein sequences for the given prokaryote analysis.

### Ortholuge analysis pipeline

The input parameters for Ortholuge include a list of tentative ortholog species groups with sets of FASTA-formatted sequences for each respective gene/protein in the tentative orthologs set. A flowchart overview of this pipeline can be seen in Figure 2. If ortholog groups have not been pre-determined, the Ortholuge software we developed is capable of calculating an initial list of tentative orthologous groups, using the RBH approach. In this latter case, the input required is a FASTA-formatted list of sequences from genes predicted in three genomes to be examined (two sequences to be compared, one reference sequence as an outgroup). Note that whole-genome data does not necessarily need to be used, however the dataset should be large enough to ensure that the distribution of relative evolutionary distances will centre around what is likely the true median for the relative evolutionary distance for the given organisms being examined.

#### 1. Sequence alignments

Initial alignments of the genes/proteins for each tentative ortholog group are generated using CLUSTALW [23] with either DNA or PROTEIN alignment options. All other parameters are default.

#### 2. Automated alignment editing

All alignment overhangs and poly-A tails are removed in each aligned set of sequences. An alignment must be aligned over 300 base pairs (bp) or 100 amino acids (aa) or it is discarded from the analysis. This choice of threshold was based on previous studies that have suggested that it is more likely that a sequence codes for a protein if its length is over 100 amino acids [24].

Gap masking is performed to remove ambiguously aligned gap-flanking regions. A sample of RBH-predicted ortholog sequence sets were examined to identify both gaps introduced by misalignments and gaps introduced through sequence insertions and deletions. Gap-masking simulations using various window length intervals were applied to the aligned sequences to establish a gap-masking approach. Our approach entails running a sliding 25-base pair window over the aligned sequences in both directions to assess gap percentages exceeding a 40% gap threshold. The window size and gap threshold were chosen such that overlapping windows exceeding the gap threshold would produce a worst-case gap masked region of 49 base pairs.

Both the trimming and gap-masking methods were evaluated for the introduction of ratio distribution biases by selected alignment characteristics. Selected characteristics of both trimmed and gap masked alignments were recorded and analyzed to determine whether the automated alignment editing process had created a ratio distribution bias for certain alignment characteristics. These characteristics included: number of aligned base pairs, identity over aligned length, identity over left and right ends, proportion of gaps over full length, proportion of gaps over left and right ends. Here we defined end length as MIN(.25 * alignment length, 150 bp/50aa). See also Figure 3.

#### 3. Sequence distances and calculation of Ortholuge ratios

The EDNADIST or EPROTDIST programs of EMBOSS [25] and PHYLIP 3.6 [26] software, respectively, were used to compute the nucleotide or protein distances. We opted to analyze our data using the Kimura distance formula due to its simplicity and computational efficiency. We used a conservative transition/transversion rate of 2 as an approximation, although studies do suggest that transition/transversion rates are context dependent [27]. All other parameters were defaults. The phylogenetic distances were used to compute the three ratios, Ratio1, Ratio2, and Ratio3, as described in Figure 2.

Ratios are then displayed manually in two forms: Histograms and as R1 × R2 plots. The ratio frequencies are enumerated for a given interval and histograms are constructed for all three ratios, visually displaying the ratio frequencies of tentative orthologous groups within a ratio of 2.5. The R1 × R2 plots are comprised of an x-y plot of Ratio1 versus Ratio2 which facilitates visualization of the full ratio distribution range (though zoomed in versions of these plots up to ratio values of 2.0 are also provided to facilitate viewing data in low ranges in this format).

### True-negative introduction analyses

#### Mean/iterative true-negative analysis

For the eukaryotic RefSeq-based RBH ortholog dataset, a selected proportion of the ortholog sets were randomly transformed to true-negative ortholog sets and then run through the Ortholuge analysis. We report here the results for a 25% transformation of the data, though other percentages were examined (data not shown). To do this transformation (introduction of true-negatives), the full set of mouse, rat, and human RefSeq sequence files were obtained from NCBI and a pairwise best-hits list was created using a pairwise blastall analysis with a 10e-4 E-value cut-off. An orthologous set was transformed to a true-negative by replacing one of the species sequences with another sequence that had a greater (next highest) BLAST expect value and which still satisfied a reciprocal and transitive best BLAST hit with the two other sequences in the orthologous set. In essence, we were removing an RBH-predicted ortholog and identifying another gene that could satisfy an RBH relationship. This essentially simulated what could happen if a gene was lost in one genome, or a genome sequence was incomplete, by removing a gene from a proposed ortholog "triple" and determining what the next RBH relationship would be for the remaining genes in the triple. Care was taken to ensure that the set of original sequences in the higher quality set being transformed initially satisfy an RBH relationship. Furthermore, the algorithm mandated that the non-orthologous replacement is not an isoform of the replaced sequence. Each transformed dataset was then run through the Ortholuge analysis. Such true-negative transformations were iteratively performed 50 to 100 times for each true-negative percentage proportion. A mean true-negative value and standard deviation for each ratio value in the distribution could then be calculated. Note that this same approach was also used to perform an iterative true-negative analysis for other eukaryotic data sets, and the prokaryotic data.

The true-negative mean and standard deviations were analyzed to establish conservative ratio cut-offs and estimate false-positive proportions. A three-level classification system for true mean false-positive values over defined ratio intervals was derived from this analysis (see "Establishing Cut-offs" Method's section, below). The number of ratios in a given set falling into each level (i.e. "probable ssd-ortholog", "uncertain", and "probable paralog" classes) was counted.

#### True-negative introduction in the bacterial set – an introduction of all possible true-negatives

For the bacterial RBH-predicted data set, *P. syringae *genes (ingroup2) were replaced with their next best reciprocal BLAST hit to *P. putida *(within a 10e-4 E value cut-off), wherever possible. 668 out of 1456 ortholog triples were transformed into true-negative triples for this dataset. There are no iterations necessary here, so the transformed data set was then run through Ortholuge once.

### Establishing cut-offs for Ortholuge-predicted "probable paralogs", uncertain, and probable ssd-orthologs

Researchers are of course encouraged to use the above true-negative analysis to formulate their own cut-offs, since cut-offs of differing levels of sensitivity and specificity are possible. In our example analysis, we examined the iterative/mean true-negative analysis for a eukaryotic and prokaryotic dataset using a histogram and examined the data in terms of the proportion of introduced true-negatives identified in each ratio range. These percentages are used to aid in identifying cut-offs for more specific (precise) identification of probable orthologs (or ssd-orthologs) and probable paralogs. We examined the trend manually, and opted to identify "probable ssd-orthologs" as those occurring in ratio ranges where there were, on average, only between 0–10% introduced true-negatives (out of the total number of tentative orthologous groups in the range; see Results, Figure 8). Tentative orthologous groups falling in ratio ranges that contained between 10 to 50% introduced true-negatives (on average) were classified as orthology "uncertain". Finally, groups falling in ratio ranges that contained greater than 50% introduced true-negatives were classified as "Probable paralog". We chose this cut-off because it was at this point that the transition from few introduced true-negatives in a range, to mostly introduced true-negatives in a range, increased significantly. Note that at this point there will also likely be some true-negatives occurring in the analyzed dataset (as illustrated also by our "higher quality" data set analyses), and so the actual proportion of true-negatives at this probable paralog cut-off point will likely be much higher. As mentioned in the results, we opted to perform this analysis on completely automated RBH data (RefSeq-based), rather than high quality data, since we appear to be able to obtain meaningful results, while being able to take advantage of the automated nature of RBH data set generation. However, we did also perform this analysis on the high quality data set, and on any EGO data set, generating comparable results.

### Identification of in-paralogs

For those tentative orthologs predicted by Ortholuge to be ssd-orthologs (and also for other classes as well, in case researchers wish to use other cut-offs), we performed an additional analysis to identify cases of in-paralogy that may affect the possible functional equivalence of the ssd-orthologs (see the Introduction for a discussion of this issue). To do this, we combined both ingroup species' sequences into one database and performed a BLAST analysis using all the individual sequences from each of the ingroup species as a query. We then identified individual sequence cases in which top hits (other than a query sequence self-hit) were to another sequence in its own species. If the bit score for this same-species hit was greater than the bit score for the other species hit, then the case was flagged as an in-paralog candidate (ie. a gene duplication may have occurred after the speciation, potentially affecting the function of the ssd-ortholog). Any such in-paralog cases were classified under the "uncertain" category, unless they had been classified, according to a Ratio1 and Ratio2 analysis, as belonging to the "probable paralog" category (in the latter case they would remain in the probable paralogs category). Note that this analysis only identifies a proportion of all cases – in particular very clear cut cases. It does not identify all possible in-paralogs and researchers are encouraged to investigate any such cases more thoroughly.

## Authors' contributions

BH and FSLB developed the initial framework for this computational method and FSLB led formation of the final draft of the manuscript. DLF and YYL developed the final methodology, performed the analyses of the selected data sets, and drafted the initial versions of the manuscript, with each focusing their research and analyses on eukaryotic and prokaryotic data, respectively, during their research rotations. FMR participated in the design of this study and provided critical input that improved this work. MRL used initial scripts developed by DLF and YYL to develop a software package that will perform the main component of the Ortholuge analysis. All authors read and approved the final manuscript.

## Supplementary Material

Additional file 1 **Supplementary Figures**. **Supplementary Figure 1**. Ratio1, Ratio2 and Ratio3 histograms of the *P. putida *– *P. syringae *– *E. coli *putative orthologous sets summarizing results of a true negative introduction analysis. **Supplementary Figure 2**. Ratio2 and Ratio3 histograms of the mouse-rat-human putative orthologous sets indicating the average proportion of true negatives observed in our simulation of an incomplete genome through the iterative introduction of a mouse (ingroup1) paralog in randomly selected ortholog sets. **Supplementary Figure 3**. Histograms of Ortholuge Ratios 1, 2, and 3 for the mouse-rat-human RBH RefSeq nucleotide dataset. **Supplementary Figure 4**. Histograms of Ortholuge Ratios 1, 2, and 3 for the mouse-rat-human OrthoMCL protein dataset. Click here for file

## Figures and Tables

**Table 1:** Ortholuge-ratios can help predict which gene in a given putative orthologous group is likely a paraloga^a^.

**Ratio1**	**Ratio2**	**Ratio3**	**Probable Paralog**
^a^			Ingroup1 paralog
	^a^	↓	Ingroup2 paralog
↓	↓	-	Outgroup paralog^b^
or ^c^	or ^c^	variable ^d^	Ingroup1 & Ingroup2 paralogs
↓	variable ^d^		Ingroup1 & Outgroup paralogs
variable ^d^	↓	↓	Ingroup2 & Outgroup paralogs

^a ^Only selected scenarios are listed. Arrows indicate relative increases or decreases in a ratio value, when compared to the highest frequency values in a histogram plot (i.e. "expected" ratio value). Smaller arrows indicate that the increase is less. In the case of the ingroup1 or ingroup2 paralog scenarios, it will depend on how divergent the paralog is and how distant the outgroup is.

^b ^Note that an outgroup paralog cannot be discriminated from cases of orthologs, nor does this analysis need to discriminate such cases (see text). However, this has been included in the table solely to illustrate how ortholog paralog cases can be discriminated (using Ratio 3) from cases where there is a combination of an ingroup1 (or ingroup2) paralog and an outgroup paralog.

^c ^This scenario will resemble an ingroup1 paralog scenario or ingroup2 paralog scenario, if one of the two ingroup paralogs diverged much more than the other.

^d ^The variation may be an increase or decrease, depending on which of the two paralogs is more diverged. Ratio 3 can help resolve such cases.

**Table 2:** Proportion of RBH-predicted^a ^orthologs that are likely ssd-orthologs^b ^and likely paralogs, according to Ortholuge analysis.

**Data set**^c^	**Probable ssd-ortholog**			**Orthology uncertain**^f^			**Probable paralog**		
	**Ratio Range**^c^	**Proportion of introduced true-negatives in a true-negative analysis**^d^	**Proportion of RBH-predicted orthologs**^e^	**Ratio Range**^c^	**Proportion of introduced true-negatives in a true-negative analysis**^d^	**Proportion of RBH-predicted orthologs**^e^	**Ratio Range**^c^	**Proportion of introduced true-negatives in a true-negative analysis**^d^	**Proportion of RBH-predicted orthologs**^e^

rat-mouse comparison (human outgroup)	R1 ≤ 0.60 and R2 ≤ 0.55	0.8%	76%	See footnote^f^	16%	14%	R1 > 0.80 or R2 > 0.80	77%^d^	10%
*P. putida-P. syringae *comparison (*E. coli *outgroup)	R1 ≤ 0.55 and R2 ≤ 0.70	1.3%	91%	See footnote^f^	24%	4%	R1 > 0.75 and R2 > 0.85	87%	5%

^a ^RBH-predicted = Predicted to be orthologous using a Reciprocal-best BLAST hit approach.

^b ^"Supporting-species-divergence orthologs" = orthologs that appear to have diverged only due to speciation and have diverged at an expected relative rate for the species. Such orthologs are likely to have more similar function. See text for details.

^c ^Ratio Range for both Ratio1 (R1) and Ratio2 (R2). See Figure 8C for a schematic illustration of the cut-off ranges on a R1 × R2 plot.

^d ^Proportion of introduced true-negatives for the 25% true-negative analysis is shown here, however the actual number of true-negatives will be higher due to false-positives likely occurring in the original ortholog dataset. This analysis was used to estimate % false predictions in range (see text and Figure 8.

^e ^RBH-predicted data sets were examined using the cut-offs generated by the true-negative analysis, to identify what proportion of all RBH-predicted orthologs fell within each range. For the rat-mouse comparison 6294 RefSeq-based groups were classified into "probable ssd-ortholog", "uncertain", and "probable paralog" classes. For the *Pseudomonas *comparison, a total of 1456 groups were classified. Note that for an analysis of the EGO-based rat-mouse data set of 19,200 groups with the same cut-offs, 76% ssd-orthologs and 16% probable paralogs were predicted (when in-paralogs were not counted, because of the lack of differentiation of gene isoforms in the EGO data set).

^f ^This "uncertain" category falls between the other two ranges and is graphically illustrated, for ease of understanding, in Figure 8C. This category follows the formula (R1 > a and R1 < b and R2 < d) or (R2 > c and R2 < d and R1 < a), where a and b are the lower and upper cut-off values, respectively, for Ratio1 (i.e. lower = cut-off for ssd-orthologs and higher = cut-off for probable paralogs), and c and d are the lower and upper cut-off values, respectively, for Ratio2. Note this "uncertain" category also contains counts of in-paralogs detected (7% of eukaryotic data, and negligible for prokaryotic data) – see text for details.
