# Supplementary material for: Improving the specificity of high-throughput ortholog prediction
Source: BMC Bioinformatics. 2006 May 28;7:270. doi: 10.1186/1471-2105-7-270 (PMC1524997; doi:10.1186/1471-2105-7-270)

Additional files – Supplementary Figures

Supplementary Figure 1. Ratio1, Ratio2 and Ratio3 histograms of the *P. putida – P. syringae – E. coli* putative orthologous sets summarizing results of a true negative introduction analysis. *P. putida* (ingroup1) paralogs were iteratively introduced into a randomly selected 25% of the ortholog groups. For each ratio two histograms are shown: The first shows the average proportion of true negatives, with dashed cut-off lines providing a boundary demarcation where the upper limit of the proportion of true negatives falls at about 10 and 50 percent, respectively. The second provides an alternate view of the data, by showing the average number of all putative ortholog groups (light shaded bars; this includes introduced true negatives) compared to the average number of true negative groups (dark shaded bars). Note that the histograms for Ratio3 do not have cut-off lines shown, since we have used a combination of Ratio1 and Ratio2 to perform any cut-off-based analysis. However, it should be noted that cut-offs could be easily generated for Ratio3 as well, and could be useful for analysis of certain types of paralog-detection scenarios.

(Supplementary Figure 1)

Ratio 1

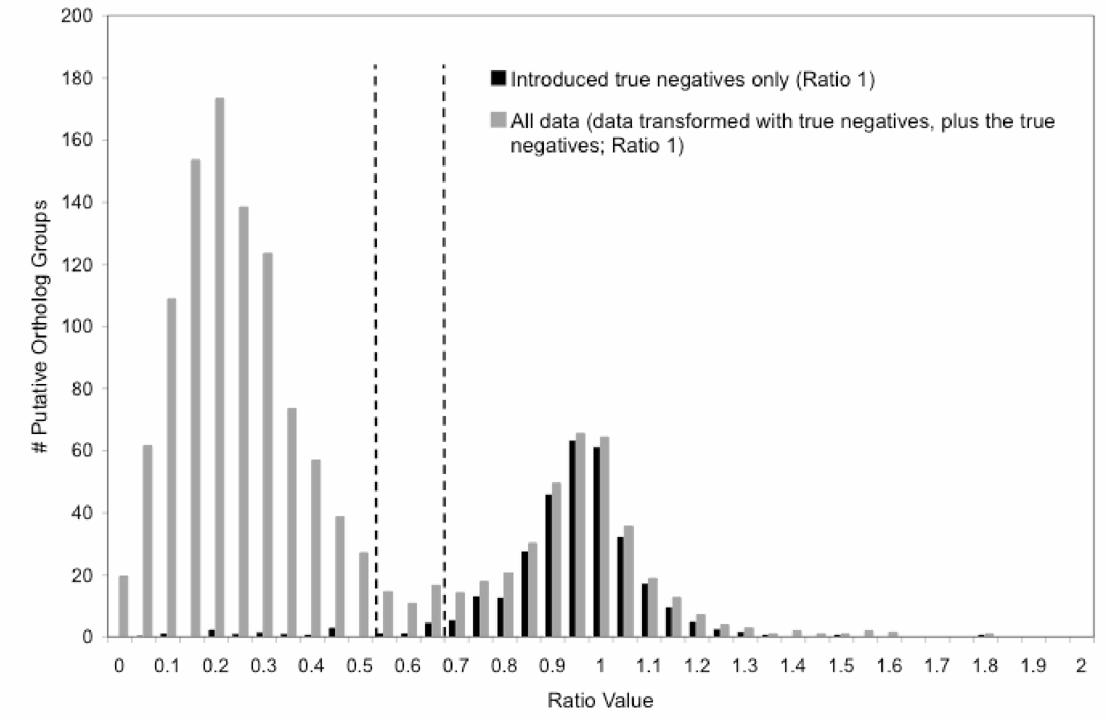

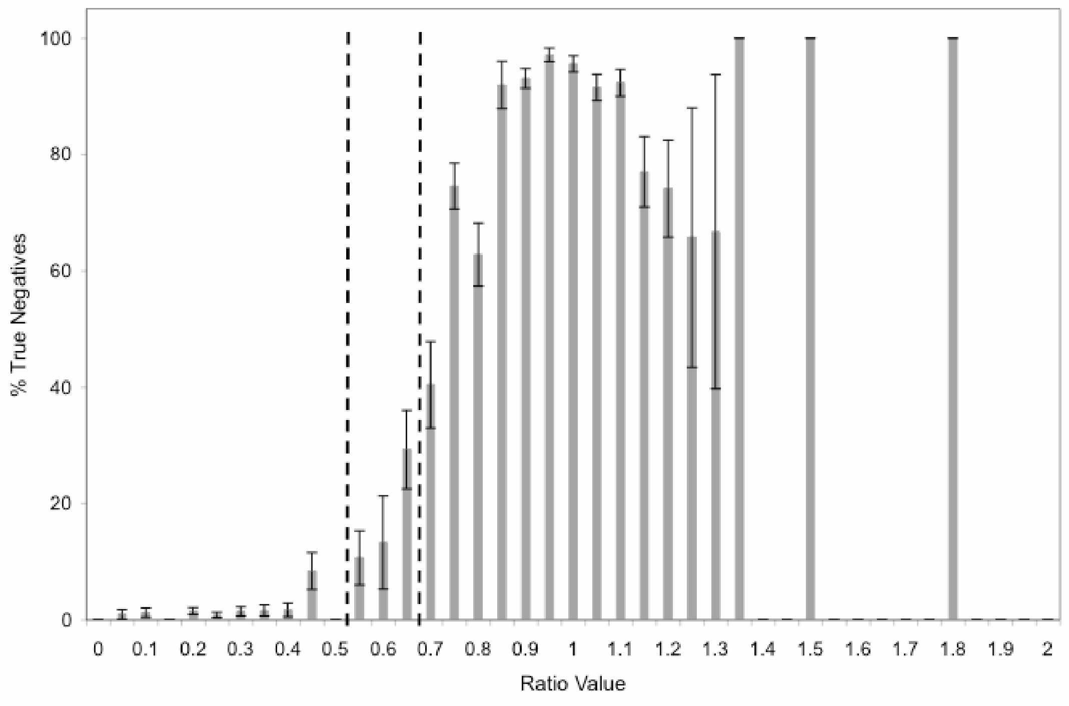

(Supplementary Figure 1 continued…)

Ratio 2

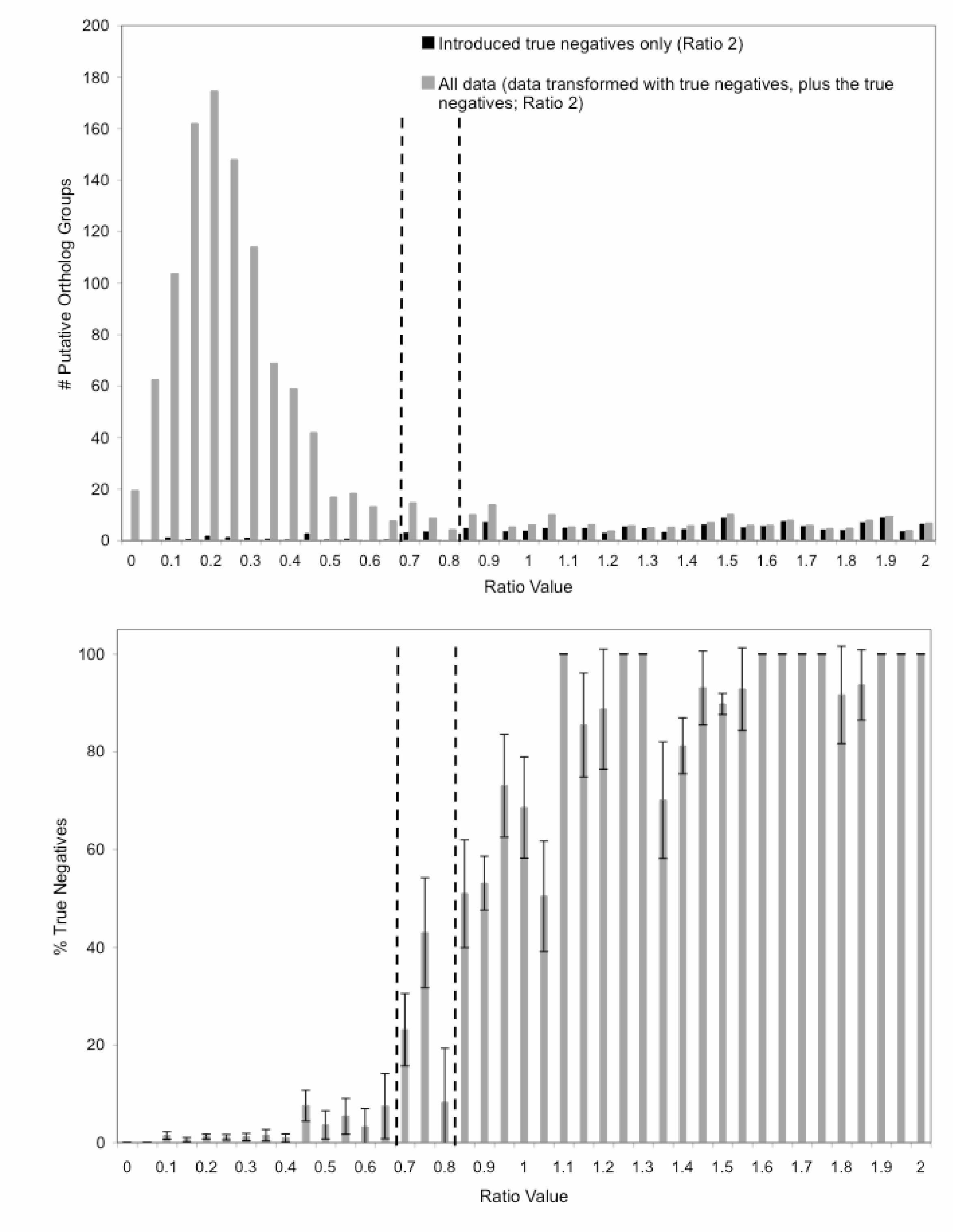

(Supplementary Figure 1 continued…)

Ratio 3

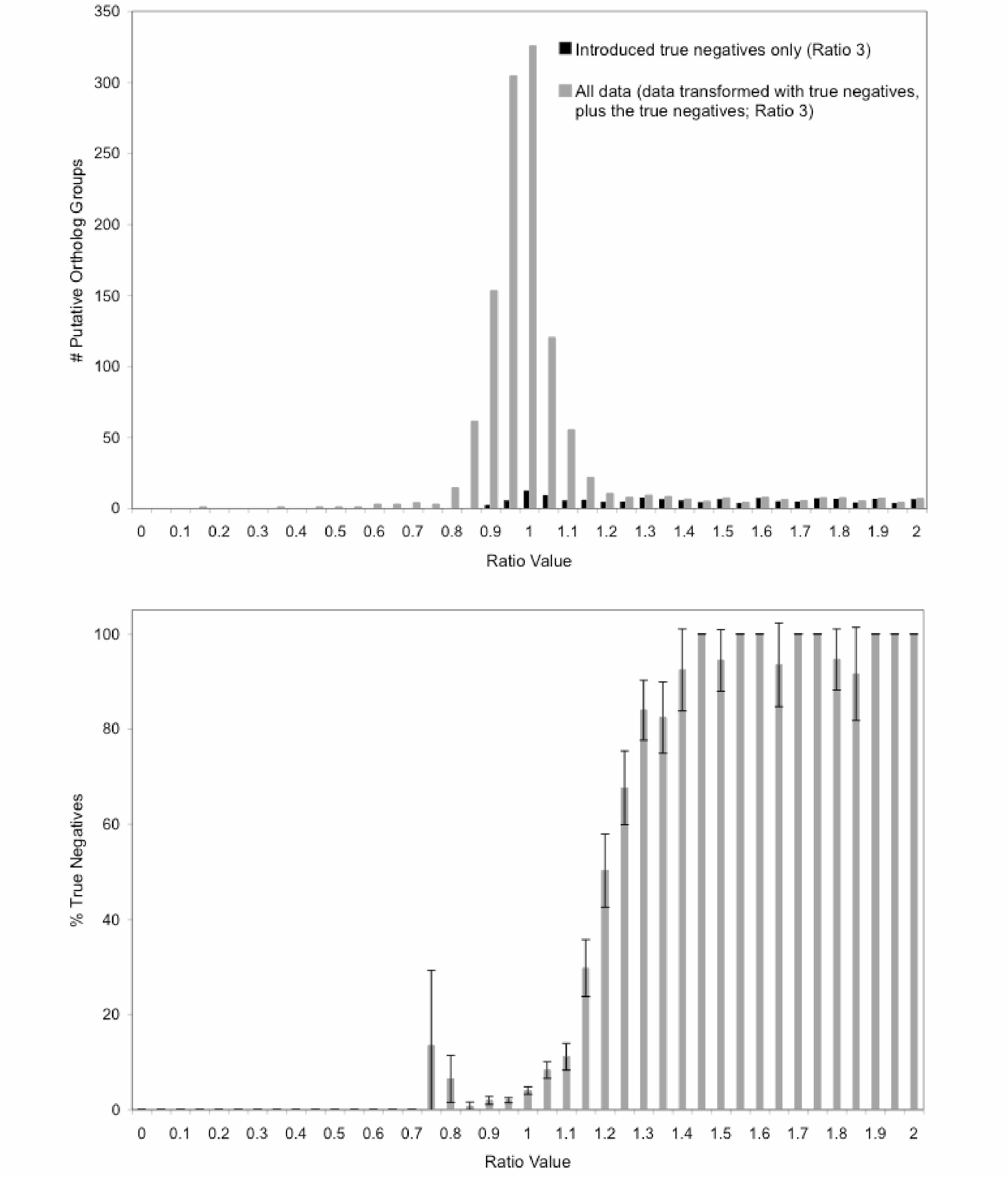

Supplementary Figure 2. Ratio2 and Ratio3 histograms of the mouse-rat-human putative orthologous sets indicating the average proportion of true negatives observed in our simulation of an incomplete genome through the iterative introduction of a mouse (ingroup1) paralog in randomly selected ortholog sets. The Ratio cut-off lines (dashed lines), provide a boundary demarcation for the proportion of true negatives falling at 10 and 50 percent, as described for the prokaryotic analysis. The corresponding Ratio1 analysis is illustrated in Figure 8.

(Supplementary Figure 2)

Ratio 2

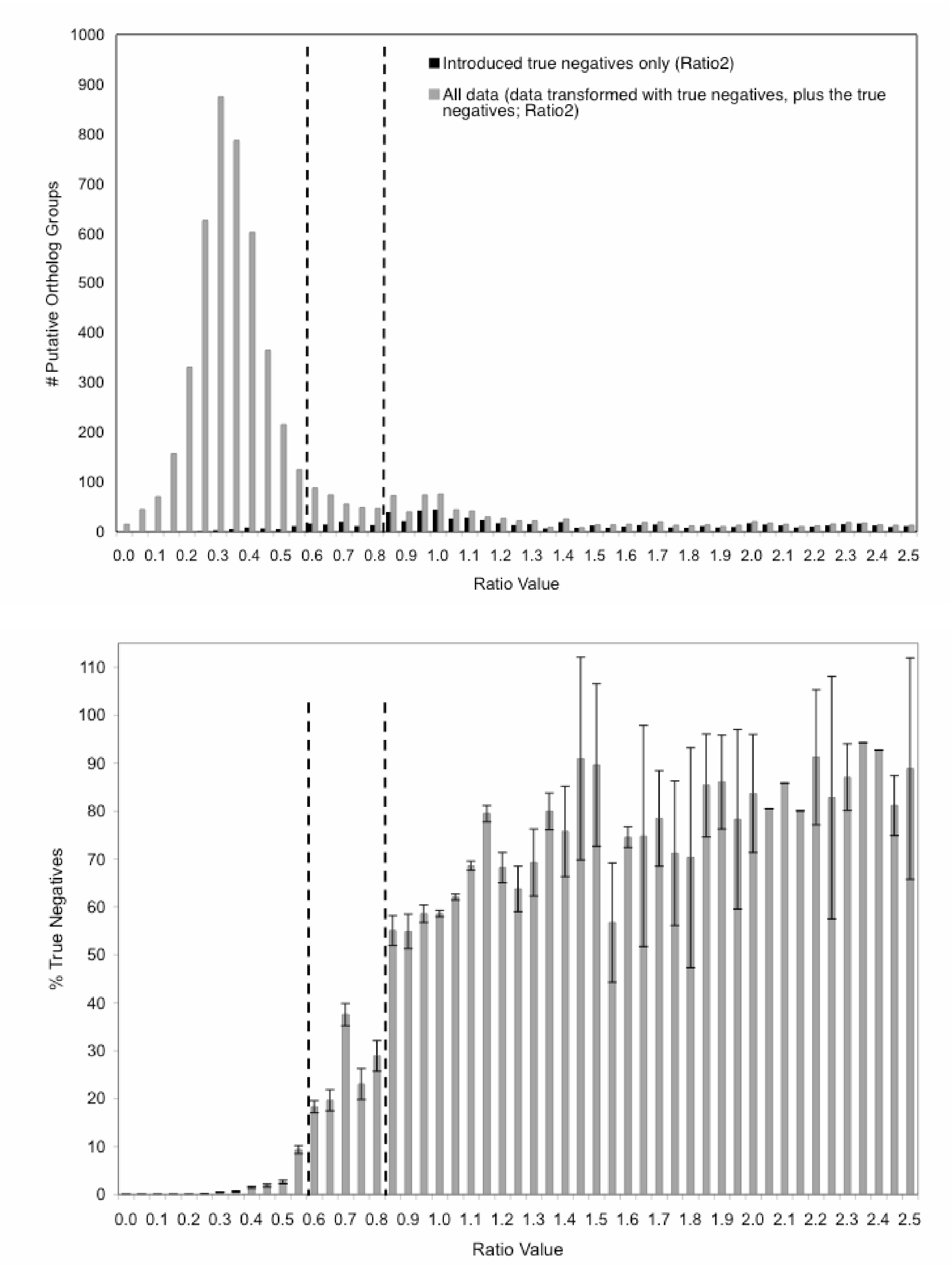

(Supplementary Figure 2 continued…)

Ratio 3

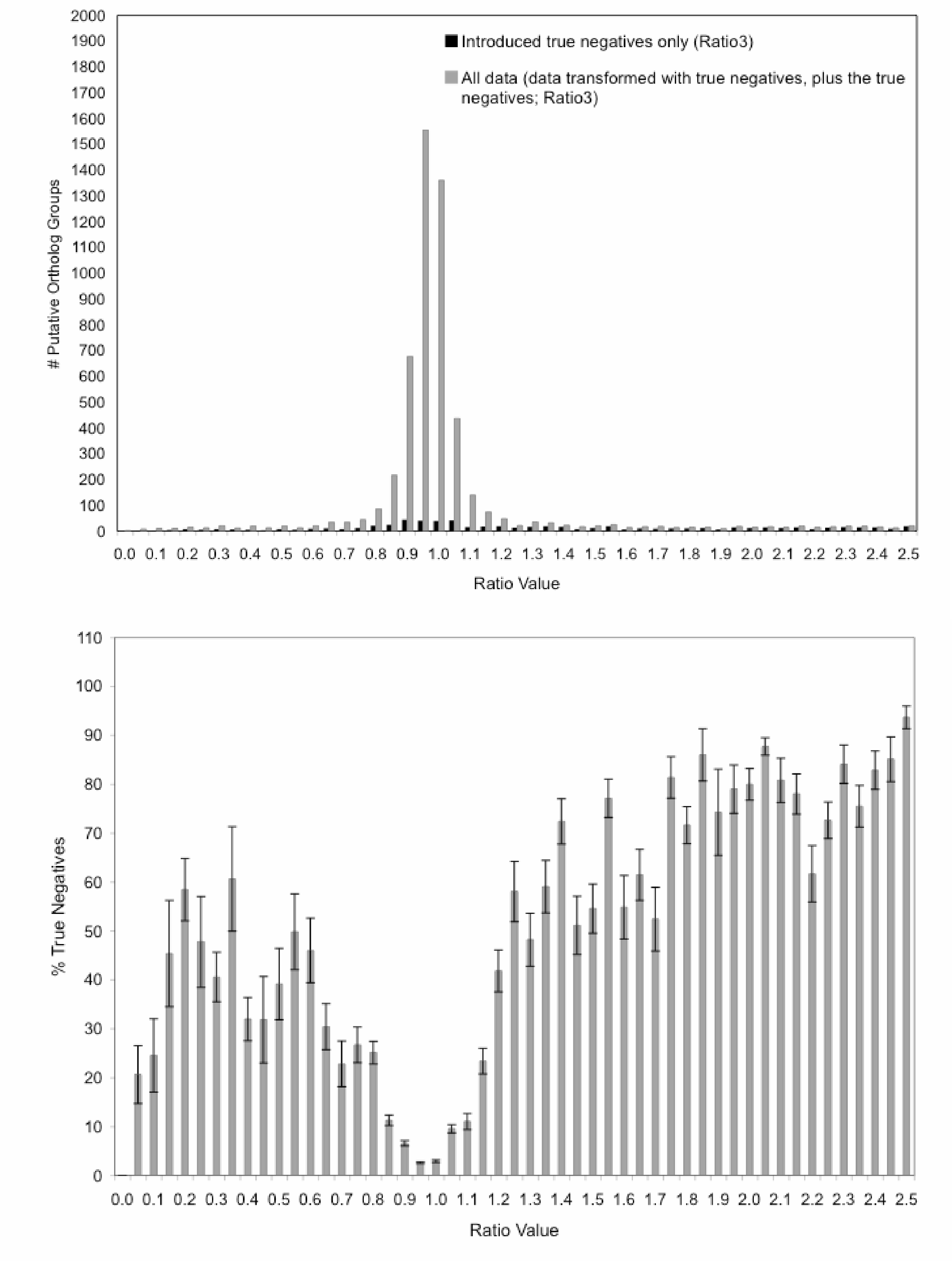

Supplementary Figure 3. Histograms of Ortholuge ratios 1, 2, and 3 for the mouse-rat-human RBH RefSeq nucleotide dataset. The mouse and rat genes were designated as ingroup1 and ingroup2, respectively. The human outgroup sequence was used to evaluate the mouse and rat evolutionary distances (see ratio functions noted in the legend). The computed ratio value frequencies are depicted in the histogram.

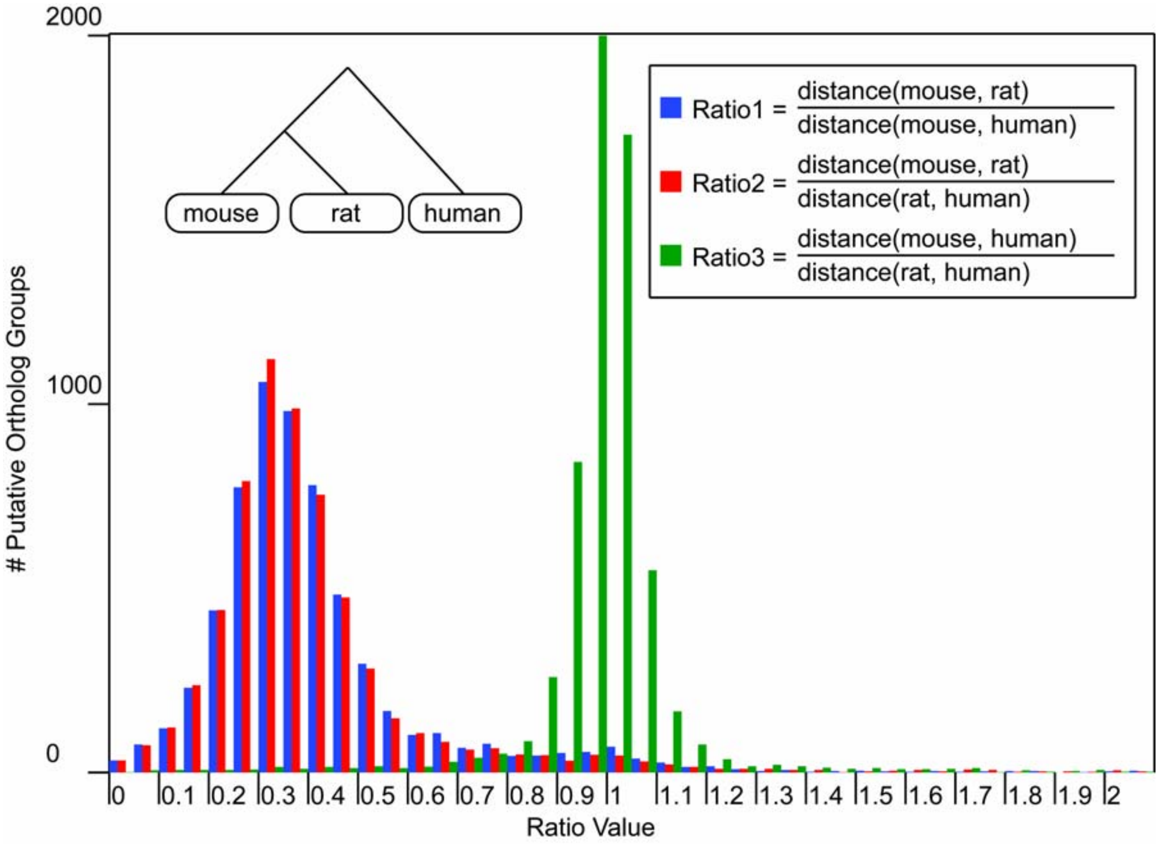

Supplementary Figure 4. Histograms of Ortholuge ratios 1, 2, and 3 for the mouse-rat-human OrthoMCL protein dataset. As in Supplementary Figure 3, the mouse and rat genes were designated as ingroup1 and ingroup2, respectively, and the human gene was designated as the outgroup sequence. The computed ratio values are shown in the histogram below. Note that this analysis was based on protein sequences, while Supplementary Figure 3 is based on nucleotide sequences. Analyses of both protein and nucleotide sequences of closely related species suggest that nucleotide sequences provide a better ratio distribution resolution. Although OrthoMCL does not use an RBH method to detect orthologs, the overall ratio distribution is similar between Supplementary figures 3 and 4. However, there is an increase in the number of sequences with Ratio1 and Ratio2 values that are around a value of 1. This is likely because OrthoMCL identifies more many-to-many orthologs that RBH methods do not detect and a proportion of these many-to-many orthologs are displaying unusual divergence. This illustrates how methods such as OrthoMCL and Ortholuge can be complementary.

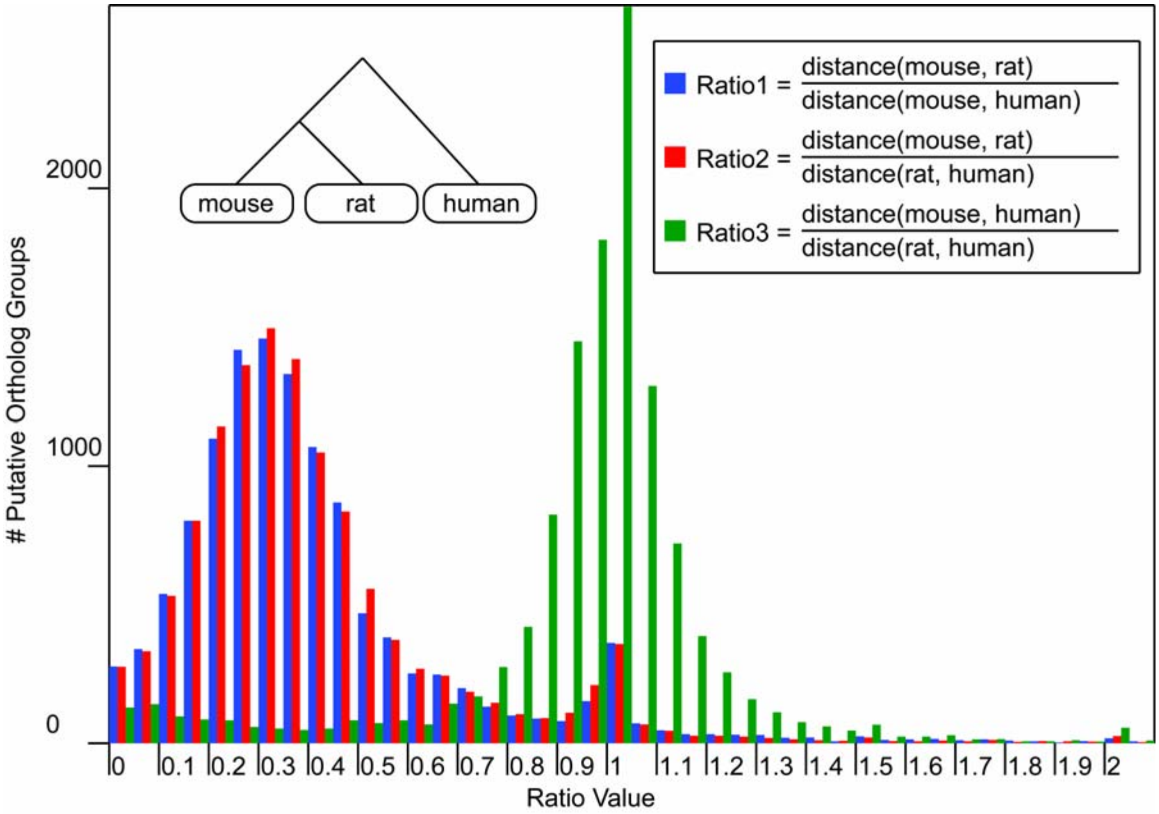

Supplement: Additional file 1 — Supplementary Figures. Supplementary Figure 1. Ratio1, Ratio2 and Ratio3 histograms of the P. putida – P. syringae – E. coli putative orthologous sets summarizing results of a true negative introduction analysis. Supplementary Figure 2. Ratio2 and Ratio3 histograms of the mouse-rat-human putative orthologous sets indicating the average proportion of true negatives observed in our simulation of an incomplete genome through the iterative introduction of a mouse (ingroup1) paralog in randomly selected ortholog sets. Supplementary Figure 3. Histograms of Ortholuge Ratios 1, 2, and 3 for the mouse-rat-human RBH RefSeq nucleotide dataset. Supplementary Figure 4. Histograms of Ortholuge Ratios 1, 2, and 3 for the mouse-rat-human OrthoMCL protein dataset. [file 1471-2105-7-270-S1.pdf]
